# A decade of commitment to hospital quality of care: overview of and perceptions on multicomponent quality improvement policies involving accreditation, public reporting, inspection and pay-for-performance

**DOI:** 10.1186/s12913-021-07007-w

**Published:** 2021-09-20

**Authors:** Astrid Van Wilder, Jonas Brouwers, Bianca Cox, Luk Bruyneel, Dirk De Ridder, Fien Claessens, Kristof Eeckloo, Kris Vanhaecht

**Affiliations:** 1 Leuven Institute for Healthcare Policy, KU Leuven - University of Leuven, Leuven, Belgium; 2 Department of Orthopedics, University Hospitals Leuven, Leuven, Belgium; 3 Department of Quality, University Hospitals Leuven, Leuven, Belgium; 4 Strategic Policy Unit, Ghent University Hospital, Ghent, Belgium

**Keywords:** Hospital, Quality improvement, Accreditation, Public reporting of healthcare data, Quality control

## Abstract

**Background:**

Quality improvement (QI) initiatives such as accreditation, public reporting, inspection and pay-for-performance are increasingly being implemented globally. In Flanders, Belgium, a government policy for acute-care hospitals incorporates aforementioned initiatives. Currently, questions are raised on the sustainability of the present policy.

**Objective:**

First, to summarise the various initiatives hospitals have adopted under government encouragement between 2008 and 2019. Second, to study the perspectives of healthcare stakeholders on current government policy.

**Methods:**

In this multi-method study, we collected data on QI initiative implementation from governmental and institutional sources and through an online survey among hospital quality managers. We compiled an overview of QI initiative implementation for all Flemish acute-care hospitals between 2008 (*n* = 62) and 2019 (*n* = 53 after hospital mergers). Stakeholder perspectives were assessed via a second survey available to all healthcare employees and a focus group with healthcare policy experts was consulted. Variation between professions was assessed.

**Results:**

QI initiatives have been increasingly implemented, especially from 2016 onwards, with the majority (87%) of hospitals having obtained a first accreditation label and all hospitals publicly reporting performance indicators, receiving regular inspections and having entered the pay-for-performance initiative. On the topic of external international accreditation, overall attitudes within the survey were predominantly neutral (36.2%), while 34.5% expressed positive and 29.3% negative views towards accreditation. In examining specific professional groups in-depth, we learned 58% of doctors regarded accreditation negatively, while doctors were judged to be the largest contributors to quality according to the majority of respondents.

**Conclusions:**

Hospitals have demonstrated increased efforts into QI, especially since 2016, while perceptions on currently implemented QI initiatives among healthcare stakeholders are heterogeneous. To assure quality of care remains a top-priority for acute-care hospitals, we recommend a revision of the current multicomponent quality policy where the adoption of all initiatives is streamlined and co-created bottom-up.

**Supplementary Information:**

The online version contains supplementary material available at 10.1186/s12913-021-07007-w.

## Introduction

Across all levels of healthcare, from micro- to macro-systems, initiatives to improve quality have been globally arising [1]. Still, patient harm continues to persist, with one in twenty patients experiencing preventable harm [2, 3] and harm putting a substantial burden on healthcare systems of high-income countries [4, 5]. Quality’s position at the top of hospitals’ agenda is therefore well-deserved.

In Flanders, the Dutch-speaking region of Belgium, a government agreement that forms the basis of today’s ‘Quality-of-Care Triad’ for the hospital setting was established in 2009. This Triad encompasses 1) voluntary announced hospital-wide accreditation, defined as an assessment of a pre-determined set of standards [6] by an international external agency, 2) voluntary measurement and public reporting of quality indicators and 3) mandatory inspection by the Flemish government. An overarching patient safety contract was drawn up at federal level between the government and acute-care hospitals from 2007, rewarding hospitals financially that committed to implementing QI initiatives with a small fixed portion of hospital payment. From 2018, the contract became known under the heading of P4P with adjusted reimbursements.

Since 2019, however, Flemish hospitals are starting to publicly express an alleged ‘quality fatigue’ [7, 8], claiming the burden of the multicomponent government policy is becoming exorbitant. However, no overview exists on how hospitals have adopted the initiatives under government policy in the past decade to corroborate this statement. Both clinicians and policymakers alike are expressing concerns on the continued application of accreditation, supported by international evidence describing it as bureaucratic and time consuming [9], merely market-driven [10], costly [11], and not promoting what actually matters to patients [12]. As a result, already about ten Flemish hospitals have declared their intention to abandon accreditation. Regarding public reporting, worries are mainly about the possibility of risk aversive behaviour in physicians that might harm patient outcomes [13], about misinterpretation or gaming of data [14], about the significant financial and administrative burden [15] and finally about the lack of reach to patients [16]. Concerning inspection, apprehension exists on the topic of ‘decoupling’, i.e. the gap between the paper-based reality of rules and guidelines and actual clinical practice [17, 18]. On the other hand, initiatives such as accreditation [19, 20], public reporting [21] and pay-for-performance (P4P) [22] have shown promise in multiple healthcare segments. Examples include accreditation promoting change and professional development [9] or public reporting further stimulating quality improvement (QI) activity and altering hospital selection by the patient [23]. This conflicting evidence urges a formal assessment on the perspectives of relevant healthcare stakeholders. Hence the objective of this study is twofold. First, to provide a detailed overview of the various initiatives that Flemish hospitals have adopted in line with current hospital policy between 2008 and 2019. Second, to study healthcare stakeholders’ perspective on the current hospital policy.

## Methods

### History of quality improvement initiatives

We conducted a retrospective region-wide multi-method study of all acute-care hospitals (*n* = 62 in 2008, *n* = 53 in 2019 after hospital mergers) in Flanders, Belgium on government-imposed QI initiatives occurring between 2008 and 2019. Information about accreditation trajectories between 2008 and 2019 was obtained from multiple sources: an online survey, Qualicor Europe (a Dutch institute focused on accreditation, formerly known as NIAZ), and public websites of hospitals. The online survey was distributed in January 2020 via Qualtrics© to all quality managers within the study sample, and contained retrospective questions about the accreditation body, the number of accreditation cycles, their audit and re-audit dates and their respective overall scores between 2008 and 2018. Secondly, data on public reporting was provided by the Flemish Institute for the Quality of Care (VIKZ), which is responsible for the measurement and the public reporting of quality indicators [24]. Thirdly, information on inspection dates and hospital mergers was obtained from the Department of Health at the Flemish government. Finally, the Federal Public Service for Health (federal government) provided information on the participants to each yearly patient safety contract between 2008 and 2017 as well as to the pay-for-performance initiative from 2018. A more detailed overview of the data collection guide and characteristics of the various QI initiatives under government policy in Flanders can be found in Additional File 1.

### Perspectives on current policy

We assessed healthcare professionals’ perspectives on current policy in two ways: a widespread online survey and an in-depth questionnaire in a focus group with Flemish healthcare policy experts. First, a survey assessing respondents’ attitudes towards current policy was distributed between July and September 2020. The survey was implemented in Sawtooth© and disseminated via email to the management of all Flemish acute-care hospitals, to government representatives and to the staff members of the Flemish Patient Association (hereafter called patient representatives). Reminders were sent with the encouragement of hospital association Zorgnet-Icuro. To further increase the number of returned surveys, survey invites were published in a medical newspaper (Artsenkrant), on social media (Facebook, LinkedIn, Twitter) and the research group’s website (www.ligb.be) and participants were encouraged to further distribute the survey link to healthcare professionals. The following eight professional groups were invited to fill in the survey: doctors, nurses, paramedics, middle management & supervisors, quality staff & executives, hospital board members, government representatives and patient representatives. The survey first pertained to how respondents perceived the implementation of an external international accreditation programme (positive, neutral, negative). Subsequently, respondents were asked to rank the ten following groups according to their importance in the determination of hospital quality policy: doctors, nurses, hospital management, quality staff & executives, middle management & supervisors, paramedics, patients & family, government, board of directors and other care providers.

Second, we invited 22 Flemish top executive healthcare policy experts for a focus group in February 2020. The group consisted of hospital board members (*n* = 7), government representatives (*n* = 6), middle management (*n* = 4), patient representatives (*n* = 3) and doctors (*n* = 2) and all made significant contributions to past or current hospital policy. The focus group was moderated by KVH and DDR, while AVW and JB acted as notetakers. The session aimed to discover what expert opinion considered as the most important aspects of current hospital policy to bring to future policy discussions. We adapted the focus group methodology [25] to generate quantitative data by introducing a Qualtrics© survey to all focus group members during the session. After a short introduction section, the survey was taken by all present focus group members (average survey time was 18 min), after which the results were discussed within the group. The survey consisted of 17 in-depth statements concerning current hospital policy (see Additional File 2) and related to the currently implemented QI initiatives, i.e. accreditation (*n* = 5), public reporting (n = 5), inspection (n = 5) and pay-for-performance (*n* = 2). The focus group members were asked to indicate how important they considered the statement to be included in future hospital quality policy discussions by means of a slider scale ranging between 0 (not important) to 100 (very important).

### Statistical analyses

For our first objective, an overview of the adopted QI initiatives was visualised. For clarity, inspection dates were grouped into ‘compliance monitoring’ and ‘other inspections’, while all individual release dates for public reporting across the four overarching domains are jointly displayed. Only the dates of the public release of indicators were presented, while data on measurement and benchmarking within hospitals were disregarded (see Additional File 1). To generate healthcare professionals’ perspectives on current policy, we first described results from the widespread Sawtooth© survey by describing the attitudes towards accreditation (positive, neutral or negative). Variation in accreditation attitudes across respondents (by one of eight invited professional groups) was assessed by means of a Kruskal-Wallis test. Data on the importance of the ten surveyed profession groups in the determination of quality policy were summarised by ranking the sum of ranks for all respondents and by invited profession (eight groups). This information was depicted by means of a radar chart, with the lowest rank representing the highest importance. Second, results from the Qualtrics© survey disseminated during the focus group were visualised in box plots ranked from highest to lowest importance for future policy discussions. Analyses were generated using SAS© software, Version 9.4 of the SAS System for Windows.

## Results

### Sample

An overview of the adoption of government-promoted QI initiatives was provided for all Flemish acute-care hospitals (*n* = 62 in 2008, *n* = 53 in 2019 after hospital mergers). Of these, 49 are general hospitals, while four are university hospitals. The online survey on the history of QI initiatives generated a response rate of 83% (*n* = 44). The number of beds per hospital ranged between 170 and 1955 with an average of 542. To assess perspectives on current policy, first, the widespread online survey targeted towards all healthcare professionals was filled in by 486 respondents. 19 had to be excluded because they could not be categorised within the eight established professional groups, resulting in a final sample of 467 respondents. Of these, the majority were quality staff & other executives (*n* = 137), doctors (*n* = 119) or hospital board members (*n* = 74). Other respondents represented middle management & supervisors (*n* = 57), nurses (*n* = 39), government representatives (*n* = 15), paramedics (*n* = 14) and patient representatives (*n* = 12). There was a balanced representation of Flemish hospitals within the surveyed sample, with an even distribution in working experience, region, type of hospital and accreditation status among respondents. Second, 17 policy experts participated in the focus group (response rate 77%) to assess perspectives on current policy. The final group consisted of hospital board members (*n* = 6), government representatives (*n* = 4), middle management (n = 4), patient representatives (*n* = 2) and one doctor.

### History of quality improvement initiatives

**Fig. 1:**
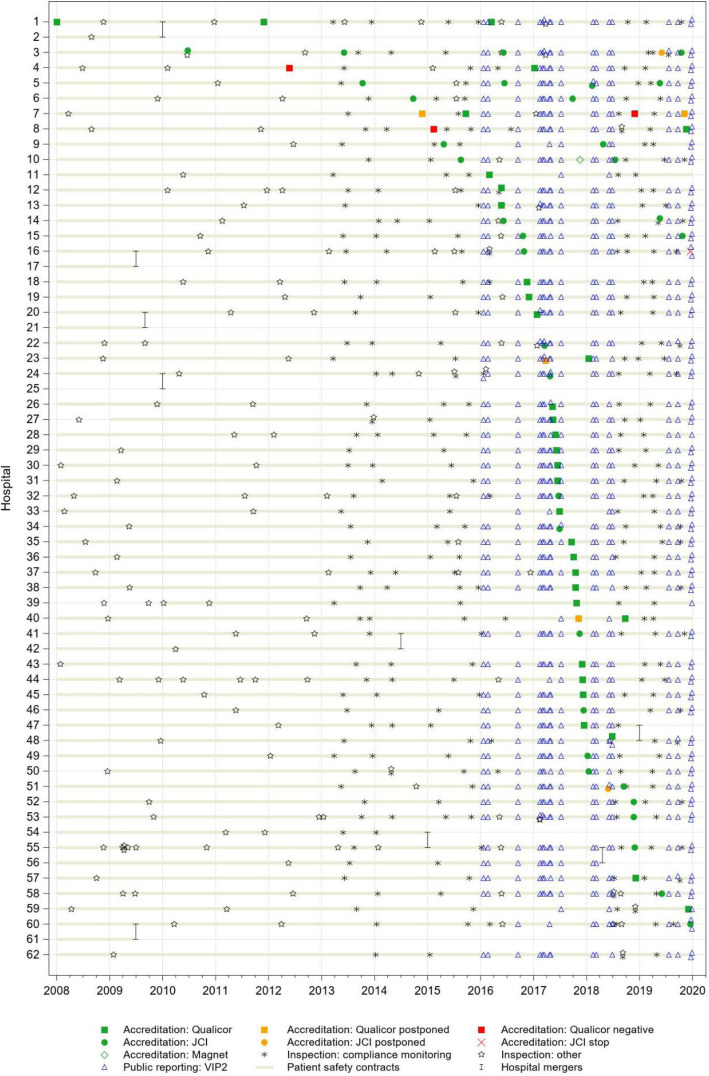
History of quality improvement initiatives in Flemish acute-care hospitals between 2008 and 2019

**Fig. 2:**
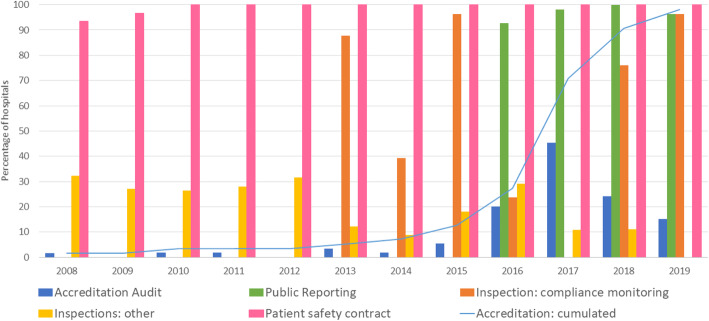
Number of quality improvement initiatives undertaken for aggregated Flemish acute-care hospitals between 2008 and 2019

Figure 1 depicts when accreditation, public reporting and inspection have taken place within Flemish hospitals and shows yearly participation to the patient safety contracts. Hospitals are ordered by date of their first accreditation audit. To date, all hospitals have entered into an accreditation trajectory by either the US-based Joint Commission International (JCI) or the Dutch Qualicor Europe (Qualicor). Only one hospital (number 62 in Fig. 1) had not yet obtained its label by the end of 2019. Few (13%) hospitals achieved their first accreditation label before 2016, but the earliest adopter (number 1) was already accredited by the beginning of 2008 and had achieved three labels by the end of 2019. The majority of hospitals opted for the four-year-cycled Qualicor accreditation (*n* = 31). JCI hospitals (*n* = 22) are audited every three years, except for the third audit in hospital 5 occurring within a year after the second due to the move to a new building. One hospital (number 10) additionally obtained a label by the US accreditation body ANCC Magnet. One hospital (number 16) opted out of the accreditation process by the end of 2019. Overall, the procurement of an accreditation label required a re-visitation in five hospitals (numbers 3, 7, 23, 40, 51) and was refused in three (numbers 4, 7, 8). Concerning public reporting, the majority of hospitals (*n* = 45) agreed to immediately start reporting from 2016 (Fig. 1). Four hospitals (numbers 10, 33, 44 and 60) waited to report their indicators until the second semester of 2016, while three started reporting from mid-2017 (numbers 11, 40, 59) and one from mid-2019 (number 39). Inspections were mostly carried out once a year, with about 30% of hospitals having inspections in 2008–2013 and nearly all hospitals from 2014 onwards. Some hospitals (e.g. numbers 22, 58) even received three inspector visits within the same calendar year, occasionally (e.g. numbers 3, 12, 14, 22, 58) concurring with accreditation visits. Finally, all but three (numbers 27, 39, 50 on Fig. 1) hospitals agreed to the federal government’s patient safety contract from 2008 (Fig. 2). By 2010, all had entered the contract.

The chances of concomitant QI initiatives have increased throughout time, as the overall number of QI initiatives across hospitals has surged, in particular in 2016 and 2017. A summary of the occurrence of initiatives per study year aggregated over hospitals can be found in Fig. 2. It demonstrates how more than 40% of hospitals received an accreditation audit in 2017, how over 90% of hospitals undertook yearly public reporting from 2016 and how inspection has monitored compliance for over 90% of hospitals in 2015 and 2019.

Table 1 provides more detailed information on the accreditation status of Flemish acute-care hospitals by the end of 2019 as well as on audit scores for each accreditation cycle. It demonstrates how the preponderance of hospitals have undergone one accreditation cycle (83%), while eight hospitals already went through re-accreditation. Accreditation details provided by 44 hospitals showed that audit scores were high on average, with global Qualicor scores ranging between 90 and 98% and the number of JCI elements not met and partially met (out of nearly 1300 measurable elements) ranging from 0 to 11 and from 0 to 43, respectively.

**Table 1:** Accreditation status in December 2019 and accreditation scores ranges between 2008 and 2018 in Flemish acute-care hospitals

Number of accreditation cycles undergone	Qualicor		JCI		
	Number of hospitals^1^	Global scores (%), range^2^	Number of hospitals^1^	Elements not met (n), range^3^	Elements partially met (n), range^3^
0	1	/	0	/	/
1	29	92–98	15	0–7	7–43
2	0	90–98	5	0–8	23–39
3	1	92–94	0	2–5	0–32
4	0	/	2	5–11	0–26

^1^Out of all 53 Flemish acute-care hospitals

^2^For 24 completed surveys

^3^For 20 completed surveys. JCI examines over 300 standards, each with their own number of measurable elements, resulting in just under 1300 measurable elements. The number displayed in this table refers to the unmet or partially met measurable elements as determined by the JCI-auditors. The exact number of standards and measurable elements varies between editions of the standards manual. In Flemish hospitals, the fourth, fifth and sixth edition of the manual were used between 2008 and 2018

### Perspectives on current policy

**Fig. 3:**
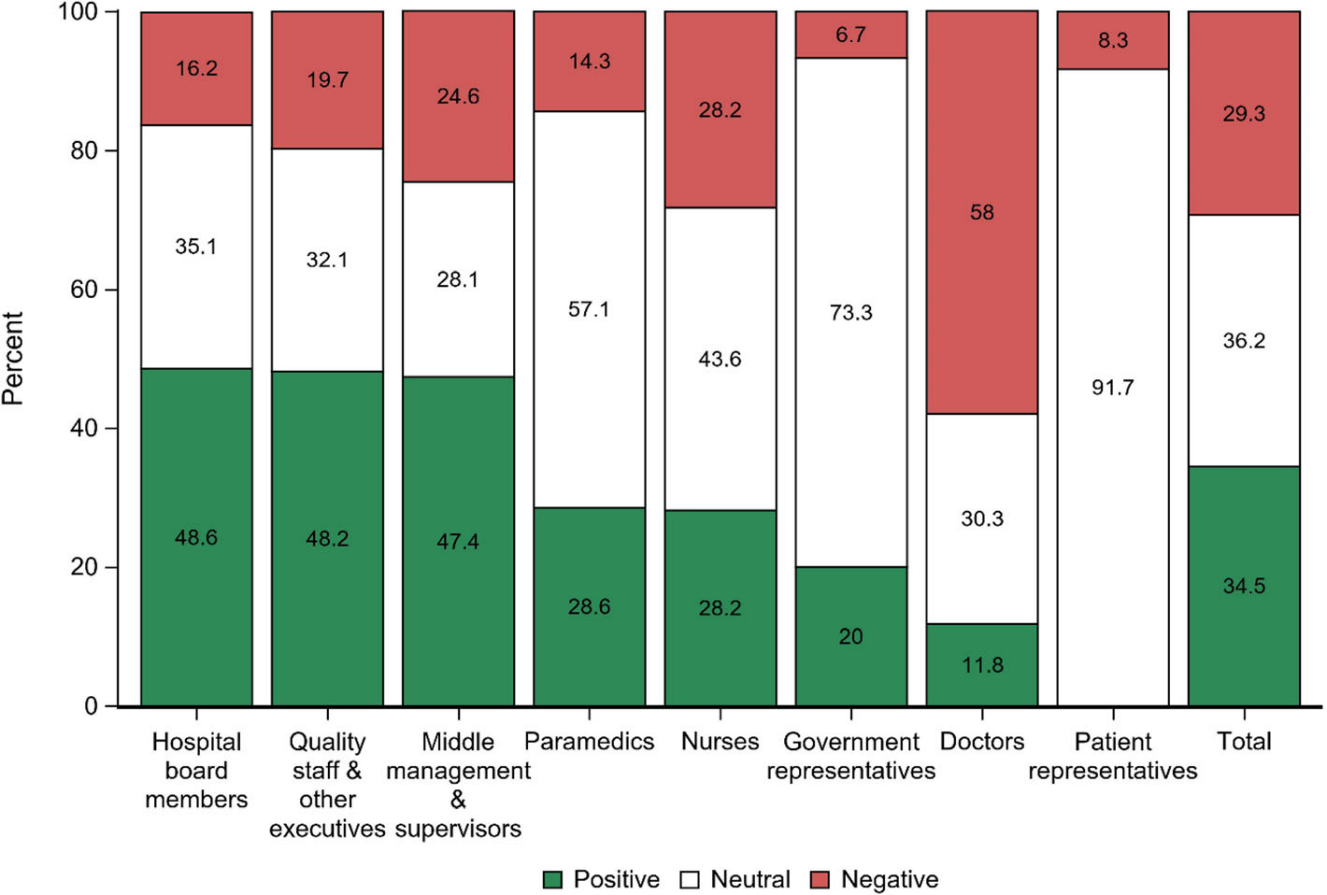
Perspectives of healthcare stakeholders on international external accreditation programmes

Figure 3 displays the perspectives of 467 healthcare stakeholders on the topic of international external hospital accreditation per profession, ranked by decreasing positive views. Overall, the majority (36.2%) of respondents had a neutral attitude towards accreditation, while 34.5% had a positive view on accreditation and 29.3% perceived it negatively. Non-clinical hospital staff were more positive about accreditation than other professional groups, with nearly half of the hospital board members (48.6%), quality staff & other executives (48.2%) and middle management & supervisors (47.4%) rating accreditation as positive. Among nurses, paramedics, government representatives and patient representatives, the majority of respondents were neutral about accreditation (43.6%, 57,1%, 73,3 and 91.7% respectively). As much as 58% of doctors had a negative attitude towards accreditation. The observed differences among professional groups were significant (p = <.0001).

**Fig. 4:**
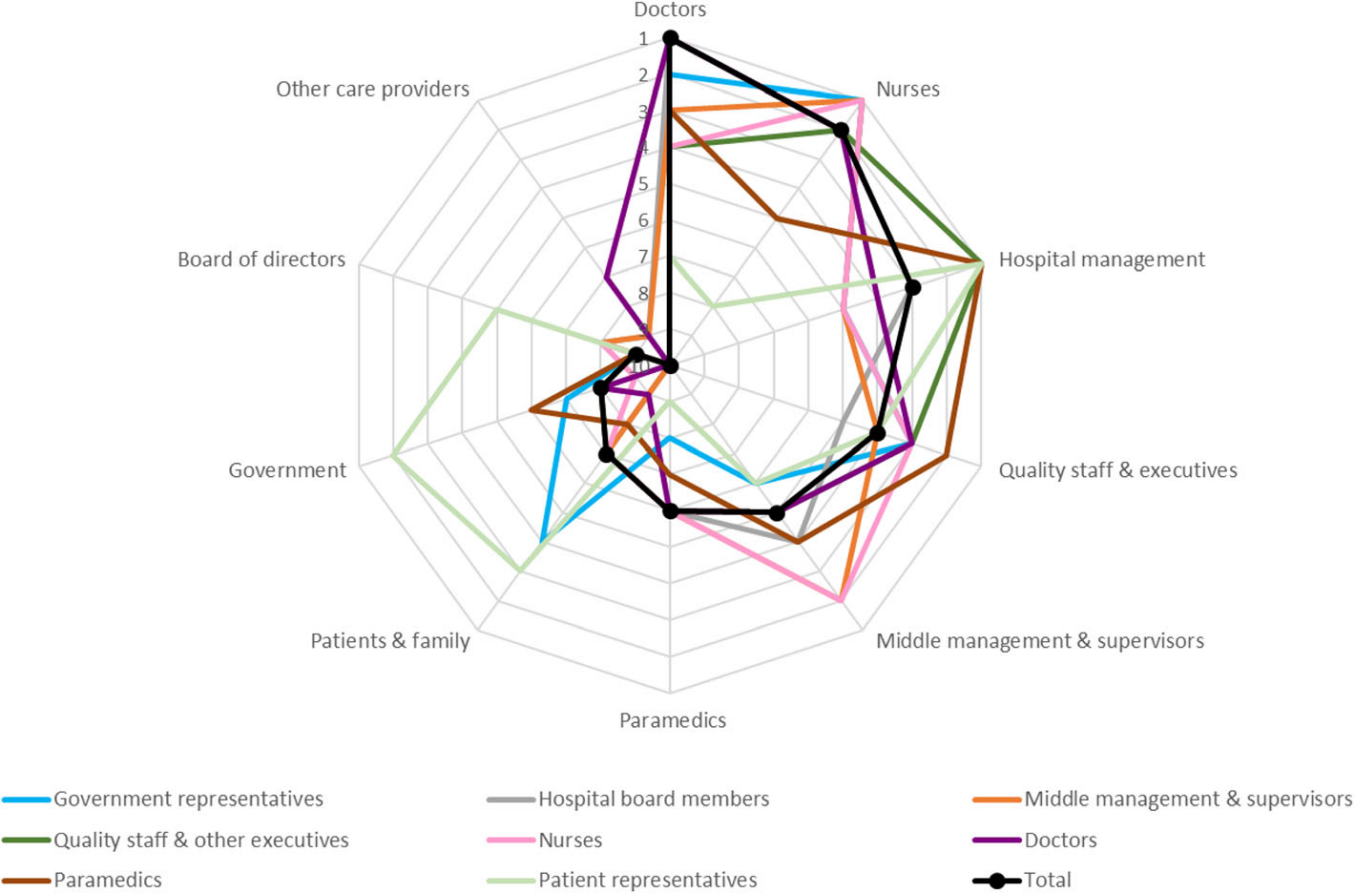
Radar diagram of healthcare stakeholders’ rankings on the importance ten professional groups have in the determination of quality policy, with the lowest ranking representing the highest importance

Overall, respondents of the online survey (*n* = 467) ranked doctors as the group with the highest importance for the determination of hospital quality policy, followed by nurses and hospital management (Fig. 4). Other care providers, government and board of directors were ranked as least important. However, different views could be observed when looking at specific types of respondents. Patient representatives, for example, found clinicians to be of minimal importance for policy setting, while they considered hospital management, government and patients & family most important. In turn, doctors found patients & family the least important contributors to quality policy. Alternatively, nurses, government and middle management & supervisors found nurses to be most important to determine policy, while quality staff & executives, patient representatives and paramedics ranked hospital management in the top position.

**Fig. 5:**
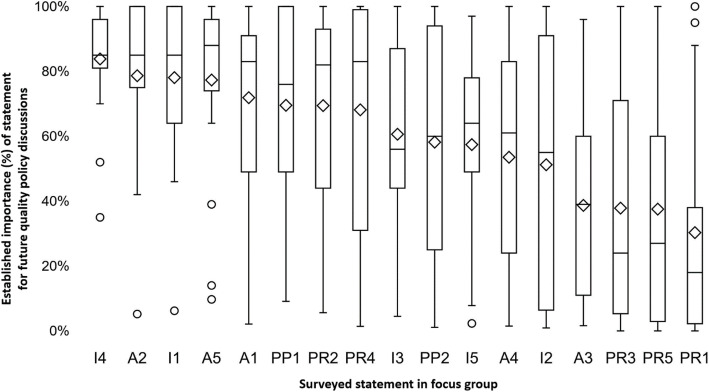
Established importance of surveyed statements for future quality discussions among focus group participants

The focus group revealed large disagreement among policy experts (Fig. 5) as there was a larger than 80% difference among the minimum and maximum range in established importance for future policy discussions in 13 out of 17 surveyed statements. Examples without concordance included the impact of accreditation on time for patient care (A3) and the involvement of mystery patients in future inspections (I2). The largest consensus as well as highest ranked importance among focus group members existed for two inspection and two accreditation statements, i.e. that inspection should focus on a minimum set of requirements (I4) and occur unannounced (I1) and that accreditation has brought about a positive dynamic within hospitals (A2) and has opened up conversation on quality within hospital boards (A5). The introduction of a minimum set of quality requirements (I4) was found most important (average importance 84%) to take to future quality policy discussions. On this topic, one focus group member stated: *“When considering to discontinue accreditation, we should be aware not to throw out the baby with the bathwater. Accreditation has opened up conversation on the topic of quality and ensured a base level we can build up from. This minimum quality level should be guaranteed in future policies.”* In contrast, the concept of patient selection and risk-avoidance by physicians in public reporting (PR1) was found least important (average 30%) to bring to future discussions, followed by the topic of public reporting on physician-level (PR5 and PR3). One focus group member discoursed the topic as follows: *“Public reporting on a physician-level is irrelevant in today’s hospital landscape. Patient care is no longer a single individual’s merit, but always involves team effort.”*

## Discussion

To our knowledge, this is the first attempt at a region-wide overview of external QI initiatives. Strengthened by its multi-method approach, our research has recapitulated paramount quality strategies implemented by hospitals between 2008 and 2019, as encouraged by the government, as well as established healthcare professionals’ viewpoint on said strategies.

This study showed that substantial commitments were made into the improvement of hospital quality in the past decade. The majority of hospitals have demonstrated they highly prioritise quality, with all hospitals opting in to the pay-for-performance programme and over 90% of hospitals actively choosing for the public reporting of quality indicators and quality assurance via accreditation. The new inspection programme focusing on patient trajectories has further stimulated this tendency towards quality by enforcing all hospitals to regularly acknowledge organisations’ current quality level. A recent surge in the implementation of accreditation, public reporting and inspections could be observed, in particular for accreditation from 2016 onwards. This growing investment into QI by acute-care hospitals is commendable. However, our research also highlights an incremental strain put on hospitals as initiatives stimulated by authorities are becoming more frequent and occasionally even concurrent. Despite all described initiatives being jointly encouraged by the government, they appear to be regarded as separate initiatives with their adoption not coordinated. This might have contributed to the alleged feeling of ‘quality abundance’ among hospital staff. To assure quality of care remains a top-priority for acute-care hospitals and current workload is reduced, we encourage a more streamlined and synchronised implementation of future quality improvement initiatives. Furthermore, this study has focused solely on external and government-encouraged QI initiatives. Coordination of initiatives should also include the supplemental initiatives hospitals have adopted internally on both patient-, department- and hospital-level, exemplified by the initiatives instigated within the domain of patient experiences [26].

Today, in the wake of the first termination of one hospital’s accreditation trajectory by an external body in December 2019, already about ten hospitals have declared their intention to abandon accreditation [8]. One potential reason for this decision might be that accreditation has failed to show distinctiveness among hospitals, with every hospital now having entered an accreditation trajectory and accreditation scores being high for all. With the large majority of hospitals also opting in to public reporting and P4P, hospitals hoped to differentiate themselves by accreditation. This distinction was encouraged by the government, as P4P points were rewarded to accredited institutions and systemic inspections were waived after entering an accreditation trajectory. However, being accredited today is no longer an assurance of competing among top-performers, it is now merely an indication of being a participant in the game, making being accredited a less coveted status to achieve prestige. Instead, accreditation has laudably provided a solid baseline level of quality for all hospitals, by ensuring they all comply with a large set of healthcare standards. Despite some doctors’ negative attitudes towards accreditation being voiced loudly within printing press [7, 8], our study consequently revealed only a minority (29.3%) of healthcare stakeholders viewed accreditation negatively. Within the focus group of policy experts, rare agreement existed on the positive dynamics accreditation have brought to hospitals. These results are in line with international findings that described overall hospital staff’s attitudes towards accreditation as positive [27, 28], with more scepticism found among physicians [27]. The latter corresponds with our finding of 58% of doctors perceiving accreditation negatively. Our study exposed a gap between clinical and non-clinical hospital staff in terms of perspectives on current policy, with clinicians most frequently displaying a negative stance towards accreditation and non-clinical staff such as hospital board, management and quality staff demonstrating a more positive attitude. While a disproportionate distribution in workload might partly explain this gap, illustrated by the fact that doctors were overall considered to be the largest contributors to quality, this also further confirms the existence of the concept of ‘decoupling’. As previously described for inspections [17, 18], a paper-based reality of rules and guidelines in the boardroom is not always reflected within clinical practice. Even among top executive policy experts within the focus group, where one would assume congruity, disagreement dominated. There is therefore a need for future policies to be co-created by all stakeholders involved, i.e. government, non-clinical staff, clinicians and patients [29, 30]. Patients in particular were found unimportant by most stakeholders and doctors especially in the determination of current policy. This is unacceptable in an era that finally acknowledges the added value of patient involvement and shared decision making in qualitative healthcare [31] and therefore urges policy change. Too often, QI initiatives have been considered as universal all-purpose solutions that work regardless of context, leading to poor fidelity and the disregarding of lessons learnt from local settings [32]. It is time quality policy was built bottom-up from clinical practice, rather than imposed top-down, making sure everyone involved can intrinsically claim ownership over quality of care.

To move forwards in the development of future healthcare policies, we recommend further research in a number of fields. First, we need stronger evidence concerning the benefits of currently employed QI initiatives. Current knowledge remains scarce and equivocal and the symbiotic effects of compound initiatives is a neglected area of research at present [33]. Minimum criteria should be determined such as a minimal set of accreditation cycles or requirements imposed by inspections. Contrastingly, maximum criteria should also be examined. Perhaps attempting more than two accreditation cycles is genuinely excessive and without additional benefit as is suggested by Devkaran et al [34]. Perhaps new policies should be considered where other high-potential initiatives should move to the forefront like disease-specific [35] or unannounced [36] accreditation or peer-review [37]. Some hospitals have already independently adopted these initiatives. We would recommend future research in the least labour-intensive way to avoid additional strain on hospital workers and management, preferably on objective data such as patient outcomes out of electronic healthcare records or discharge data sets. From the increasing adoption of QI initiatives demonstrated in this paper, it can be concluded there is a need to establish priorities for future policy, where evidence-based targets could facilitate a more coordinated and integrated policy implementation. Second, the cost of current and future employed initiatives should be assessed, to determine the further feasibility of the quality policy. QI efforts today are primarily funded by the hospitals themselves, with no additional funds provided by the government besides a limited portion of hospital finances through P4P. Policymakers should consider increasing funding for evidence-based QI initiatives. Investing in quality might result in a positive return-on-investment and at the very least could relieve some of the current pressure on hospitals and help facilitate a level of investment that can leave a durable impact on the quality of hospital care. Third, the support of the entire healthcare sector, from clinicians to hospital management to patients, should be considered for both current and potential elements of a future quality policy and a broad consensus should be strived for. As such, policy will move more towards a healthcare service that’s endorsed by both patient and healthcare provider [38, 39]. Finally, we stress the importance of a sustainability assessment of quality policy. Our paper has demonstrated the significant and increasing commitment hospitals have made in recent years. This raises questions on how much we should demand of our hospitals and especially what the threshold is above which we have asked too much. With the Covid-19 pandemic having shaken healthcare at its very core, there’s potential for rethinking current quality practice and policy from the ground up, inclusive of all stakeholders involved.

A number of considerations that merit further attention and highlight a number of limitations to this study needs to be outlined. First, results derived from the survey on QI implementation might have suffered from a response and recall bias. As primarily objective data were procured from a survey with a commendable response rate of 83% and combined with objective data from other sources, we feel this bias is minimalised to the extent possible. Second, the survey on perspectives of healthcare stakeholders did not contain questions on other specific initiatives such as e.g. governmental inspections or public reporting. Perceptions on accreditation were specifically surveyed because accreditation programmes appeared most strongly connected to feelings of dissatisfaction within hearsay and due to hospital statements claiming accreditation abandonment. Our focus group with policy experts instead focused on all government-encouraged QI initiatives and revealed large disagreement on all initiatives. As stated above, additional research is required that takes all potential initiatives and all healthcare stakeholders into account and looks for a balanced compromise. Additionally, the widespread survey generated lower sample sizes in specific groups, e.g. patient representatives. Still, those representatives constitute over a thousand patients among several patient organisations and the overall response of 467 healthcare stakeholders is laudable. Finally, our research remains limited to initiatives stimulated by government policy. The inclusion of initiatives instigated by individual hospitals might have provided a more comprehensive historic overview of QI initiatives. Nevertheless, our focus on government-encouraged initiatives exposed a disconnect between policymakers and clinicians which future policy will need to resolve, while capturing the essence of quality improvement within Flemish hospitals in the past decade.

## Conclusion

Acute-care hospitals in Flanders, Belgium, have demonstrated an increased implementation of government-encouraged quality improvement initiatives over the past decade. From 2016 onwards, the adoption of accreditation, public reporting, pay-for-performance and inspection has surged and has demanded an incremental commitment. Our study revealed healthcare stakeholders were incongruous in their viewpoints on current policy. While doctors are overall considered as most crucial in quality policy, current accreditation programmes are frequently perceived negatively by them. Nonetheless, overall views on accreditation were predominantly neutral or positive among different healthcare stakeholders. With growing concerns on the sustainability and efficacy of today’s multicomponent policy, we recommend a thorough policy revision with both patients’ and all relevant stakeholders’ involvement that prioritises and streamlines the implementation of future quality improvement initiatives.

## Supplementary Information

**Additional file 1.** Data collection guide for requested variables concerning government-encouraged quality improvement initiatives along with their characteristics.

**Additional file 2.** Statements surveyed to focus group.

## Data Availability

The datasets used and analysed during the current study are available from the corresponding author on reasonable request.

## Abbreviations

JCI: Joint Commission International
P4P: pay-for-performance
QI: Quality Improvement
Qualicor: Qualicor Europe
VIKZ: Flemish Institute for the Quality of Care
