# Supplementary material for: A decade of commitment to hospital quality of care: overview of and perceptions on multicomponent quality improvement policies involving accreditation, public reporting, inspection and pay-for-performance
Source: BMC Health Serv Res. 2021 Sep 20;21:990. doi: 10.1186/s12913-021-07007-w (PMC8450175; doi:10.1186/s12913-021-07007-w)
Supplement: Supplementary file 1 — Additional file 1. Data collection guide for requested variables concerning government-encouraged quality improvement initiatives along with their characteristics. [file 12913_2021_7007_MOESM1_ESM.docx]

**Additional File 1**

Supplemental Table 1: Data collection guide for requested variables concerning government-encouraged quality improvement initiatives along with their characteristics

| **Characteristics** | **Data sources for requested variables** |
| --- | --- |
| **General: Hospitals in Flanders** | |
| - 62 acute-care hospitals in 2008 - 53 acute-care hospitals in 2019 - 9 hospital mergers took place between 2008-2019 - Anno 2019:   - 4 university hospitals and 49 general hospitals   - Number of beds ranges between 170 and 1955   - Average number of beds: 542 | - Hospital characteristics (e.g. number of beds, teaching status): [www.health.belgium.be](http://www.health.belgium.be) - Hospital mergers: <http://atlas.ima-aim.be/databanken> |
| **Accreditation** | |
| - Voluntary. - Hospitals opting for accreditation are exempt from one part of inspection process (see below). - No national hospital-wide programme exists. Hospitals can opt for any recognised international accreditation body. - Announced - Promoted since 2009. | - For Qualicor-accredited hospitals:   - Survey dates for all audits and re-audits between 2008 and 2019   - Edition of accreditation manual   - Accreditation scores   - Status of accreditation label (achieved, postponed or declined) - Information derived from Qualicor Europe after approval of each individual hospital provided in the Qualtrics^©^ survey sent out to quality managers of all 53 hospitals. - For JCI-accredited hospitals:   - Survey dates for all audits and re-audits between 2008 and 2019   - Edition of accreditation manual   - Accreditation scores   - Status of accreditation label (achieved, postponed or declined) - Information derived from Qualtrics^©^ survey sent out to quality managers of all 53 hospitals. - For hospitals who did not respond to the Qualtrics^©^ survey sent out to quality managers of all 53 hospitals. (n=9)   - Accreditation body   - Survey dates for all audits and re-audits between 2008 and 2019 - Information derived from publicly available hospital websites [not disclosed here to safeguard anonymity] |
| **Public reporting** | |
| - Voluntary for each indicator. - Includes validated structure, process and outcome indicators across four overarching domains:   - Cancer (breast cancer, rectum cancer and lung cancer survival)   - Patient experiences   - Patient safety (hand hygiene, patient identification, medicine prescription completeness and safe surgery checklist)   - Website content - Measurement and internal benchmarking were introduced in 2013. The reporting to the general public started in 2016. | The Flemish Institute for the Quality of Care (VIKZ) provided the following information:   - Participating hospitals to the measurement and internal benchmarking of each quality indicator within the 4 domains per year (2013-2019) - Participating hospitals to the public reporting of each quality indicator within the 4 domains per year (2013-2019) - For each quality indicator: dates of measurement, availability of benchmark and public reporting on [www.zorgkwaliteit.be](http://www.zorgkwaliteit.be) for each semester between 2013 and 2019 (the same dates for all participating hospitals) |
| **Inspection** | |
| - Organised by the Flemish government. - Consists of:   - Compliance monitoring:     - Unannounced     - Compulsory for all hospitals     - Introduced in 2013     - Examines patient pathways, concentrating on a different pathway every two years: surgery (2013-2014), internal medicine (2016) and cardiology (2018-2019), with a repeat inspection for surgery and internal medicine in 2018.   - Systemic inspection:     - Announced     - Compulsory except for accredited hospitals     - Includes intensive self-assessments and risk analyses to study quality guarantees on the long term   - Safety audits:     - Unannounced   - Inspections for the purpose of allocating hospital beds:     - Announced | The Department of Health (Flemish Government) provided the following information:   - Dates of compliance monitoring surveys, systemic inspections, safety audits and allocation inspections for all Flemish acute-care hospitals between 2008 and 2019. - Hospital mergers occurring between 2008-2019 missing from <http://atlas.ima-aim.be/databanken> |
| **Patient safety contracts / Pay-for-performance** | |
| - Voluntary - A first contract was introduced in 2007 and asked for a yearly commitment between 2007 and 2012. The contract was built on three pillars: patient safety management system, transmural care and indicators. - A second contract for the period 2013-2017 focused on four general themes (safety management, leadership, communication, patient and family empowerment) and four specific themes (high-risk medication, safe surgery, transmural care, restrictive measures in psychiatric care). The criteria were determined based on international accreditation requirements to further support hospitals opting for an accreditation trajectory. - Hospitals entering the contract received a predominantly fixed budget after meeting the terms of the contract. - From 2008, the patient safety contract initiative was dismantled for acute-care hospitals and changed into a Pay-for-Performance initiative. Herein, hospitals are rewarded when they have demonstrated to have provided qualitative care. A variable budget, totaling to about 5 million on a total budget of 6.4 billion euros (Federal Public Service Health. Pay for performance-programma 2018 voor algemene ziekenhuizen. 2018) is rewarded depending on the indicators met. Indicators include hospital-wide structure and process indicators (e.g. accreditation achieved, patient experiences) as well as disease-specific process indicators (e.g. antibiotics prophylaxis). | The Federal Public Service for Health (federal government) provided the following information:   - Participating hospitals per year to the patient safety contracts between 2008 and 2017 - Participating hospitals per year to the pay-for-performance programme between 2018 and 2019. |
