# Supplementary material for: A decade of commitment to hospital quality of care: overview of and perceptions on multicomponent quality improvement policies involving accreditation, public reporting, inspection and pay-for-performance
Source: BMC Health Serv Res. 2021 Sep 20;21:990. doi: 10.1186/s12913-021-07007-w (PMC8450175; doi:10.1186/s12913-021-07007-w)
Supplement: Supplementary file 2 — Additional file 2. Statements surveyed to focus group. [file 12913_2021_7007_MOESM2_ESM.docx]

**Additional File 2**

Supplemental Table 2: Statements surveyed to focus group

| **Question number** | **Statements asked to focus group** | **Related quality improvement initiative within hospital policy** |
| --- | --- | --- |
| A1 | Every hospital should undergo a minimum of two external hospital accreditation cycles. | Accreditation |
| A2 | Accreditation trajectories bring about a positive dynamic concerning the ‘hospital quality’ mindset. | Accreditation |
| A3 | Accreditation trajectories are responsible for a decrease in time for patient care. | Accreditation |
| A4 | Accreditation trajectories are responsible for an increase in quality and middle management staff. | Accreditation |
| A5 | Discussions and actions on quality policy by hospital board members are triggered by accreditation trajectories. | Accreditation |
| PR1 | Public reporting has led to doctors selecting healthier patients. | Public reporting |
| PR2 | Data on mortality and readmission rates on a hospital-level should be made publicly available. | Public reporting |
| PR3 | Data on mortality and readmission rates on an individual physician’s level should be made publicly available. | Public reporting |
| PR4 | Data on patient outcomes such as complications and quality-of-life on a hospital-level should be made publicly available. | Public reporting |
| PR5 | Data on patient outcomes such as complications and quality-of-life on an individual physician’s level should be made publicly available. | Public reporting |
| I1 | Quality control of hospitals should involve unannounced quality checks. | Inspection |
| I2 | Quality control of hospitals should involve mystery patients to assess care quality. | Inspection |
| I3 | To assess quality of care, it is better to evaluate care programs and care trajectories than to evaluate hospital-wide quality. | Inspection |
| I4 | Every hospital should meet a set of minimum requirements for qualitative hospital care (i.e. ‘the vital few’), which are evidence-based and determined by both government and the care sector. | Inspection |
| I5 | Should a hospital achieve good quality outcomes, the quality control of its processes and protocols will become less of a priority for the inspection body. | Inspection |
| PP1 | Hospitals with good quality outcomes should be rewarded financially. | Pay-for-performance |
| PP2 | Physicians with good quality outcomes should be rewarded financially. | Pay-for-performance |
